# Traction force and tension fluctuations in growing axons

**DOI:** 10.3389/fncel.2015.00417

**Published:** 2015-10-29

**Authors:** Robert J. Polackwich, Daniel Koch, Ryan McAllister, Herbert M. Geller, Jeffrey S. Urbach

**Affiliations:** ^1^Department of Physics and The Institute for Soft Matter Synthesis and Metrology, Georgetown UniversityWashington, DC, USA; ^2^Developmental Neurobiology Section, Cell Biology and Physiology Center, National Heart, Lung, and Blood Institute, National Institutes of HealthBethesda, MD, USA

**Keywords:** growth cone, stress, mechanical, traction force, axon outgrowth, DRG neurons

## Abstract

Actively generated mechanical forces play a central role in axon growth and guidance, but the mechanisms that underly force generation and regulation in growing axons remain poorly understood. We report measurements of the dynamics of traction stresses from growth cones of actively advancing axons from postnatal rat DRG neurons. By tracking the movement of the growth cone and analyzing the traction stress field from a reference frame that moves with it, we are able to show that there is a clear and consistent average stress field that underlies the complex spatial stresses present at any one time. The average stress field has strong maxima on the sides of the growth cone, directed inward toward the growth cone neck. This pattern represents a contractile stress contained within the growth cone, and a net force that is balanced by the axon tension. Using high time-resolution measurements of the growth cone traction stresses, we show that the stress field is composed of fluctuating local stress peaks, with a large number peaks that live for a short time, a population of peaks whose lifetime distribution follows an exponential decay, and a small number of very long-lived peaks. We show that the high time-resolution data also reveal that the tension appears to vary randomly over short time scales, roughly consistent with the lifetime of the stress peaks, suggesting that the tension fluctuations originate from stochastic adhesion dynamics.

## 1. Introduction

Cell growth and movement is an inherently mechanical process, involving modulation of intracellular and extracellular forces, but surprisingly little is known about the role of dynamic forces in axon growth and guidance (Franze et al., 2013). Axons are under tension during growth and tension is actively regulated *in vivo* (Rajagopalan et al., 2010), and growth rates can be modulated by externally applied forces (reviewed in Suter and Miller, 2011). Changes in tension have also been shown to affect vesicle transport and synapse formation (Ahmed and Saif, 2014). During outgrowth, the tension is related to traction stresses generated by force-bearing adhesion sites between the growth cone and the extracellular matrix (ECM). We have recently shown that mapping of those stresses provides a dynamic readout of neurite tension, as well as a detailed picture of the complex pattern of stresses generated during growth (Koch et al., 2012). Similar results were obtained in studies of *aplysia* bag cell neurons (Hyland et al., 2014).

As summarized below in the Background section, the fluctuating forces in the axon and growth cone involve the orchestrated activity of a diverse array of cytoskeletal components and motor proteins. The assembly and disassembly of structures linking the cytoskeleton to the substrate and the generation of active forces is an inherently stochastic process. The cell must manage these random fluctuations to produce sustained growth, and adjust those same processes as needed to produce changes such as growth cone turning in response to guidance cues. The fluctuations themselves may provide clues to the mechanism of orchestration. In this manuscript, we investigate several interrelated aspects of force fluctuations revealed by traction force microscopy: the persistent stress patterns that underlie the complex traction stresses generated by the growth cone, the lifetime distribution of local stress peaks, and the dynamics of fluctuations in axon tension. Taken together, these results support a model of force dynamics where motor activity in the axon and growth cone is continually generating contractile stress, and the observed fluctuations originate primarily from the stochastic assembly and disassembly of adhesions in the growth cone.

## 2. Background

Axon growth and guidance is controlled by the complex interplay of actin polymerization, actin retrograde flow, and microtubule polymerization (reviewed in Dent et al., 2011; Gomez and Letourneau, 2013; Liu and Dwyer, 2014). Actin polymerization generates lamellipodial protrusions and filopodial growth at the leading edge of the growth cone, and adhesion sites link the actin cytoskeletal to the ECM. This process is catalyzed by guidance cues and growth-promoting ligands that trigger signaling that locally modulates actin polymerization (reviewed in Suter and Miller, 2011; Gomez and Letourneau, 2013). Adhesions, also called focal or point contacts, act as a “clutch” which, when engaged, produce a reduction in myosin-II powered retrograde flow that can facilitate membrane protrusion and microtubule invasion into the peripheral zone of the growth cone (Chan and Odde, 2008; Gomez and Letourneau, 2013). The local stresses generated by the growth cone thus provide a direct dynamic readout of clutch engagement (Chan and Odde, 2008; Koch et al., 2012; Toriyama et al., 2013; Hyland et al., 2014).

By generating stresses through ECM linkages, cells can sense the stiffness of their environment, and there is now extensive evidence that the mechanics of the cellular environment plays a critical role in a wide range of normal and pathological biological processes, and that neuronal response to rigidity plays a role in both normal development (Tyler, 2012; Franze, 2013) and the effectiveness of implants intended to promote recovery from nervous system injury (Minev et al., 2015). The brain and spinal cord are extremely soft (Franze et al., 2013), so the rigid glass substrates used in most *in vitro* studies provide a particularly poor representation of the *in vivo* environment. Neuronal growth and dendrite branching are modulated by substrate stiffness (reviewed in Franze et al., 2013), and some of the mechanosensing pathways have been identified (Previtera et al., 2010; Kilinc et al., 2014), but the picture remains mostly incomplete.

We have shown that DRG neurite outgrowth is optimal on substrates of intermediate stiffness while hippocampal neuron outgrowth appears insensitive to stiffness, and that this difference in sensitivity is correlated with differences in adhesion-generated traction forces (Koch et al., 2012). Stress-generating adhesions provide the critical mechanical link between the growth cone and the ECM, and thus are central players in rigidity sensing, tension generation and the modulation of cytoskeletal dynamics during neurite outgrowth and guidance. The production of traction stresses represent one central target of the richly complex biochemical signaling pathways associated with motility and guidance (Dent et al., 2011), and conversely the stress generation produces distinct signaling from adhesion proteins (Kuo, 2013). Stress fluctuations therefore represent an invaluable readout of the growth cone's rigidity sensing and motility machinery.

## 3. Materials and methods

### 3.1. Cell culture

All animal experiments in this study were conducted in accordance with the recommendations of the Institutional Animal Care and Use Committee (IACUC) of Georgetown University, using a protocol approved by the IACUC. Dorsal Root Ganglia (DRG) were obtained from the lumbar region of P0-P1 rat pups, trimmed, washed in Dulbecco's modified Eagle's medium (DMEM), and enzymatically digested for 20 min in 3 ml 0.25% trypsin/10 μg/ml DNase/Ca^2+^ and Mg^2+^-free Hanks balanced salt solution (HBSS). The reaction was stopped by an addition of an equal volume of fetal bovine serum (FBS), followed by an addition of DMEM to a final volume of 15 ml. Ganglia were then dissociated by titration with a fire-polished Pasteur pipette. Cells were then pelleted, resuspended in 5 ml DMEM, and passed through a 100 μm cell strainer. The cell strainer was rinsed twice with 5 ml DMEM and the cell suspension was pooled, pelleted, twice washed in DMEM, and resuspended in Neurobasal media (Life Technologies, Grand Island, NY). Cells were cultured in Neurobasal medium with 2% B27, 5% horse serum, 100 units/ml penicillin, 100 mg/ml streptomycin, 0.5 μg/ml Fungizone, and 10 mM HEPES along with an addition of 2 nM nerve growth factor. Cells were plated at relatively low densities of 1 × 10^4^ cells/dish and incubated at 37° and 5% CO^2^ atmosphere. Glia cell numbers were kept very low by careful trimming during the dissection process and plating at low density. Furthermore, single neurons/growth cones were selected for observations to avoid interference from glial cells or other neurons. Cell viability on the microscope stage was ensured by means of a live cell chamber (Tokai Hit, Shizuoka-Ken, Japan) equipped with an objective heater that controlled temperature and pH.

### 3.2. Polyacrylamide hydrogel substrates

Polyacrylamide (PAA) hydrogels were prepared according to published procedures (Pelham and Wang, 1997; Sabass et al., 2008), with some modifications. Briefly, 20 mm glass-bottomed dishes (MatTek, Ashland, MA) were wiped with 0.1 N NaOH and silanized with 3-aminopropyl-trimethoxysilane (Sigma-Aldrich, St. Louis, MO). The dishes were washed extensively and the glass surface was treated for 30 min with 0.5% glutaraldehyde followed by a final wash. Afterwards, 18-mm coverglasses were coated with Sigmacote (Sigma-Aldrich) to make non-adhesive top coverslips. Acrylamide and bis-acrylamide (Bio-Rad Laboratories, Richmond, CA) were mixed in PBS solution to a final volume of 1 ml at appropriate concentrations to achieve the desired gel stiffness. For traction force measurements, FluoSphere bead solution (0.2 μm, 660 nm; Invitrogen) was added at 5% volume. The final solution was degassed for 15 min and put on ice for 5 min. Polymerization was initiated by addition of 10 μl freshly prepared ammonium persulfate (10% w/v solution; Sigma-Aldrich) and 3 μl of *N,N,N,N*-tetramethylethylenediamine (TEMED; AcorsOrganics, Morris Plains, NJ). Immediately after initiation, 5 μl of PAA solution was pipetted onto the MatTek dish coverglass and the non-adhesive top coverslip was quickly placed onto the gel droplet and gently pressed down. The dish was inverted to facilitate settling of fluorescent beads at the upper gel surface. After 30 min, the gel was immersed in water for 10 min, and then the top coverslips were gently removed under water. The gels were allowed to swell in dH^2^O for 1–2 h before the surface coating treatment. The gels were coated with 2 μg/cm^2^ [5 μl of 1 mg/ml CellTak (BD BioSciences) in 200 μl dH^2^O] in a 20 min incubation at room temperature followed by μg/cm^2^ (5 μl of 1 mg/ml laminin in 200 μl PBS) laminin incubation for 2 h at 37° C. Before seeding with cells, the gels were incubated in cell culture media for a minimum of 2 h at 37° C. Previous studies showed no significant difference in laminin adsorption onto gels of different stiffness (Georges et al., 2006; Kostic et al., 2007). Stiffness was characterized during polymerization by rheology measurements performed on a stress-controlled bulk rheometer (Anton Paar KG, Graz, Austria) with 1 Hz oscillatory shear at 1% strain for 30 min. The Young's modulus used in the traction stress calculation was determined from the measure storage modulus G′ as *E* = 2G′(1 +ν) using a Poisson ratio ν of 0.45 (Frey et al., 2007).

### 3.3. Image processing and traction force microscopy

PAA hydrogels were prepared as described above, and the displacements of the fluorescent beads on the gel surface were tracked in order to determine the traction stresses generated by the growth cones. (See Style et al., 2014, for an overview of this technique). Specifically, fluorescence images of beads and bright-field transmission images of cells were recorded as 3D stack time series for 1–2 h with 1–5 min time resolution on a Leica TCS SP5 confocal laser scanning microscope (Leica, Deerfield, IL) equipped with a 63x water immersion objective at *z* steps of 0.3–0.5 μm. Image slices of each stack were median filtered to reduce noise, and afterwards each stack was reduced to a 2D image via maximum intensity projection. The resulting images were drift-corrected by detecting the shift of each image with respect to the first image. The shift was calculated in each corner region (1/5 × 1/5 of the image) from the peak of the cross-correlation, and the median of the four values was used for the drift correction. The reference image representing the zero-stress configuration was calculated from the median of the intensity time course at each pixel. Bead displacements between images and the reference image were detected on a 0.75 μm 2D grid using cross-correlation. The corresponding deformation field was obtained by 2D Gaussian interpolation. We calculated the traction stress field from the deformation field for each image in a time series by implementing a Fourier transform-based algorithm using the Boussinesq Green's function as presented by Sabass et al. (2008).

In order to minimize Z-drift, high time-resolution data was taken as 2D images only after a minimum system equilibration time of 1 h with the sample dish on the microscope stage. The data was recorded with time intervals of 1 or 2 s with manual correction of Z-focus every few minutes when necessary to correct for any residual Z-drift.

### 3.4. Growth cone traction force analysis

**Figure 1:**
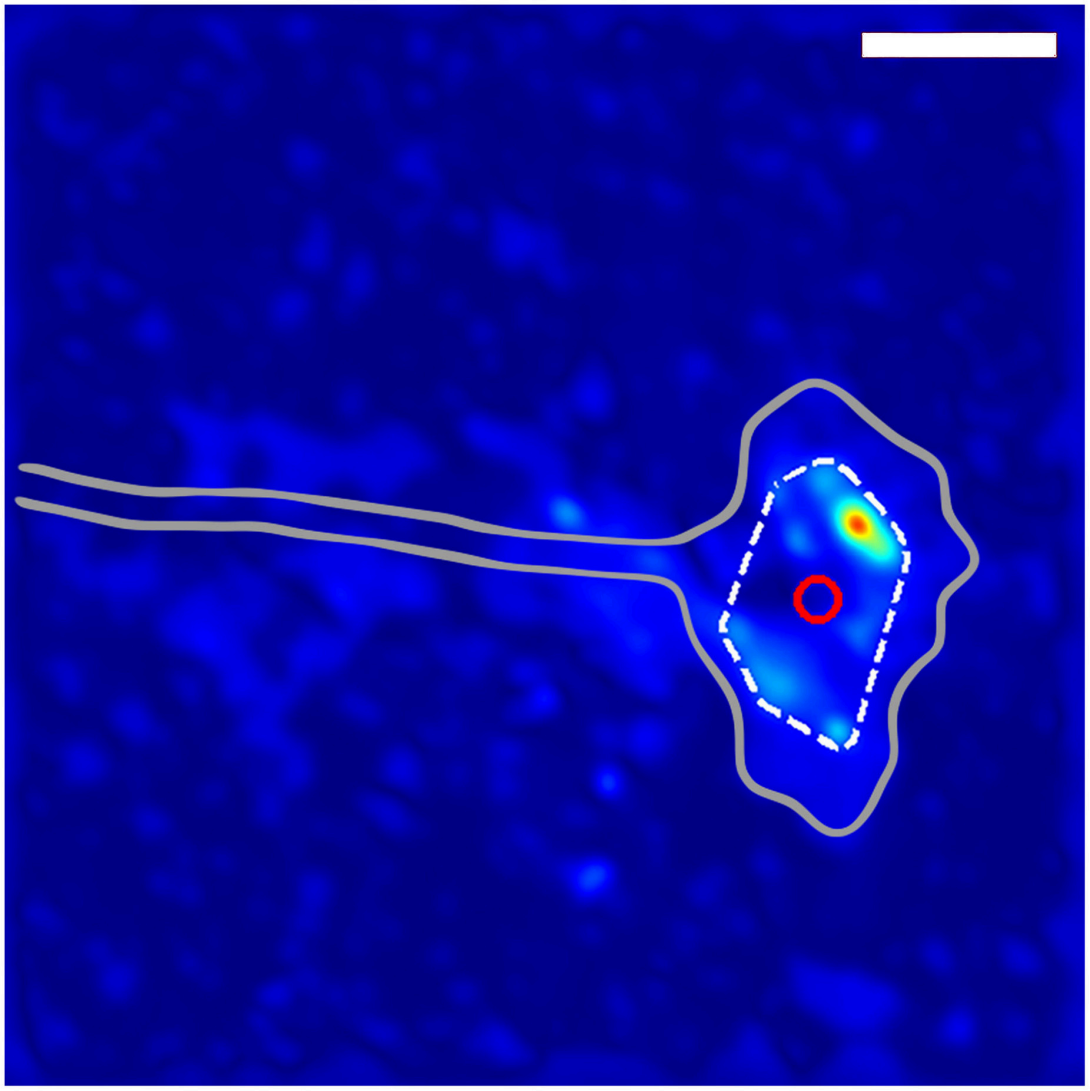
**Method for determining growth cone position**. The gray outline shows the general shape of the growth cone. The dashed while line indicates the convex hull of the thresholded traction force map, and the red circle shows the location of the centroid of the convex hull used to define the location of the growth cone. The scale bar represents 10 μm.

**Figure 2:**
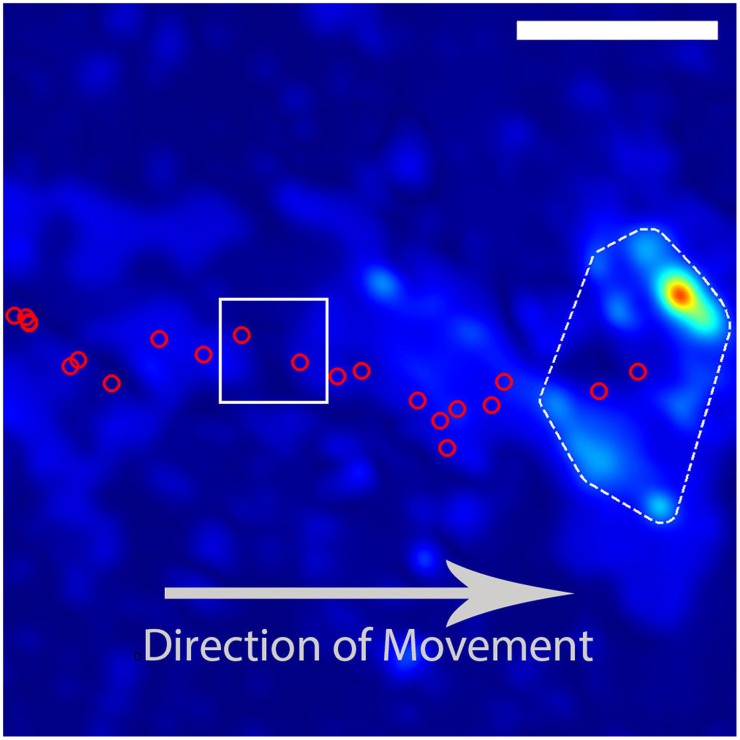
**Example trajectory of a DRG growith cone**. The red circles indicate the calculated positions of the growth cone based on images taken at intervals of 180 s. The growth cone is moving from the left to the right of the image. The DRG final position and traction stress field as depicted in Figure 1. The red circles inside the white box indicate the growth-cone positions used in Figure 3. The scale bar represents 10 μm.

Each dataset considered consisted of a set of *N* observations, or images, separated by time Δ*t*. For each image in a dataset, we calculated a relative stress threshold *S*_*threshold*_

(1) $$S_{threshold}(n)=3S_{noise}(n),$$

where we defined the stress noise, *S*_*noise*_, as the median value of the maximum stresses extracted from the four corner regions of the traction stress map associated with observation *n*. We imposed the threshold *S*_*threshold*_(*n*) on each corresponding traction stress map, resulting in at least one surviving region of stress. We then drew the convex hull defined by the stress peaks of the thresholded traction stress map, which can generally be understood as the polygon determined by the smallest perimeter totally enclosing all remaining stress regions. We defined an approximate “location” of the growth cone by calculating the centroid (geometric center) of the resulting convex hull (Figure 1). The resulting set of position vectors $R=\left\{{\vec{r}}_{1},{\vec{r}}_{2},\ldots {\vec{r}}_{n},\ldots {\vec{r}}_{N-1},{\vec{r}}_{N}\right\}$ described the trajectory of the growth cone over the timespan of the dataset (Figure 2). We defined the vector ${\vec{v}}_{n}$

(2) $${\vec{v}}_{n}=\frac{{\vec{r}}_{n+1}-{\vec{r}}_{n}}{\Delta t},$$

as the growth cone velocity, giving the instantaneous direction of travel of the growth cone at the time of observation *n*.

**Figure 3:**
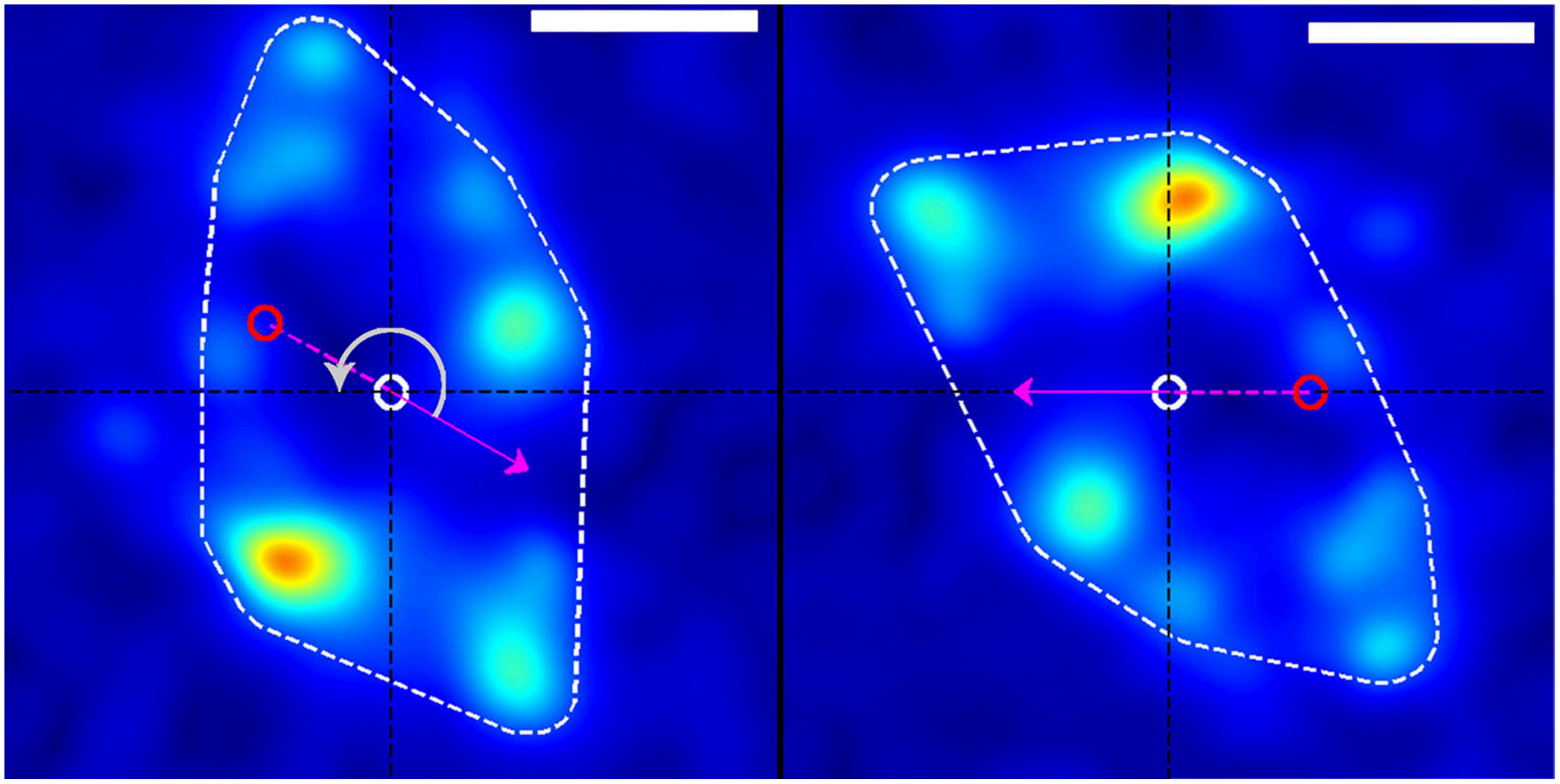
**Rotation of stress field coordinate system (left) into the frame of reference of the growth cone (right)**. The dashed white border shows the convex hull of the thresholded traction stress map from which the growth-cone position was calculated. The red and white circles represent the growth-cone positions at the previous and current time points, respectively (in this example, from the white box in Figure 1). The magenta arrow indicates the direction of movement of the growth-cone between these time points. The panel on the right is produced by rotating the stress field on the left through an angle (gray arrow) around the current centroid (white circle) such that the direction of motion is placed along the negative x-axis. This procedure transforms from the reference frame of the laboratory to the frame of reference or perspective of the growth cone. The scale bar represents 5 μm.

**Figure 4:**
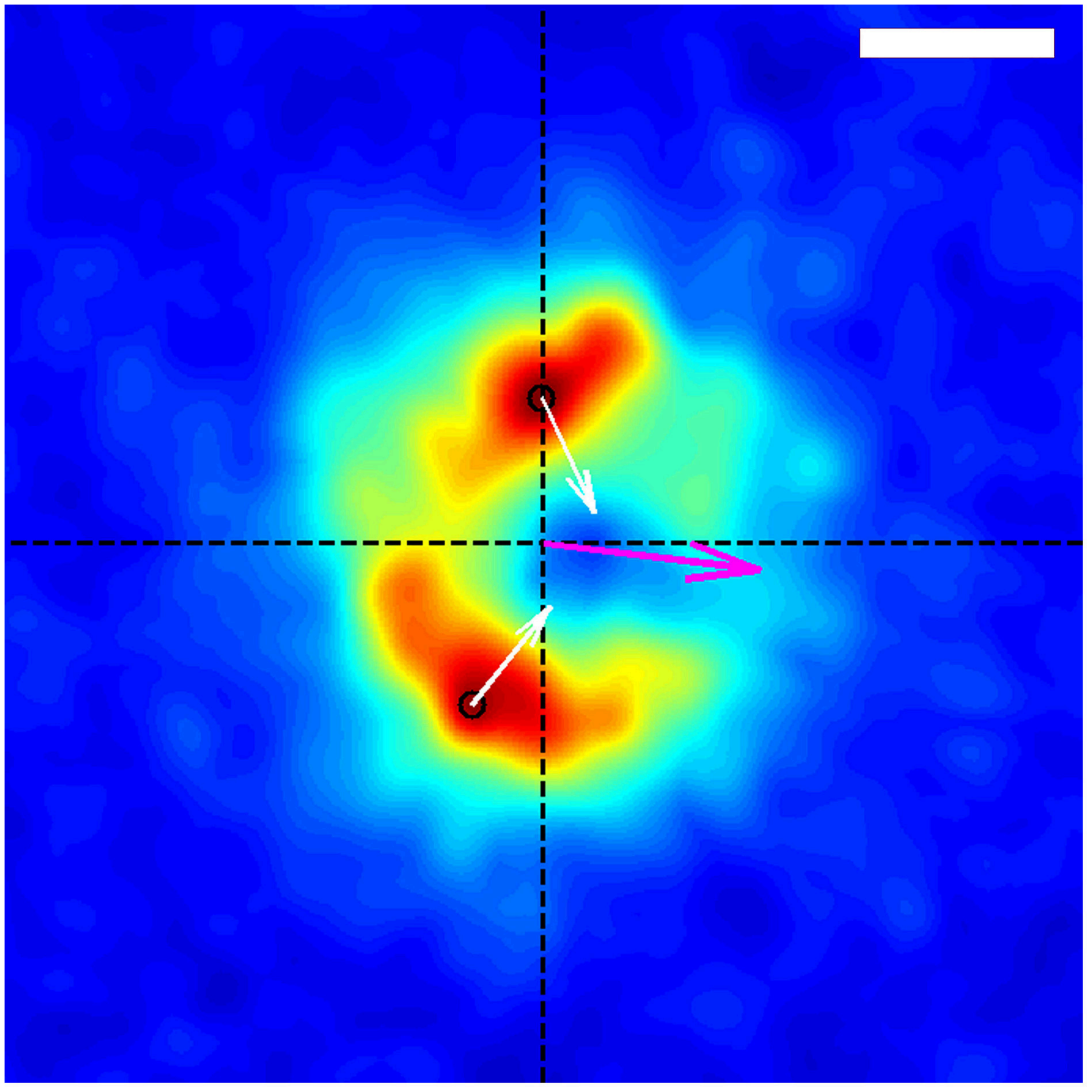
**Average spatial distribution of traction forces exerted by the growth-cone shown in Figure 1 over the time span of the entire data capture, in the frame of reference of the growth-cone**. The growth-cone is moving to the left along the horizontal axis shown. The purple arrow situated at the origin indicates the direction of the net force. The black circles indicate the locations of the stress peaks in the positive and negative y-planes, and the white arrows depict the corresponding direction and relative magnitude of those stresses. The scale bar represents 5 μm.

To investigate the nature of traction force generation from the “perspective” of the growth cone, we independently rotated each image's traction force map around the convex hull centroid so as to orient the growth cone velocity along the negative x-axis (Figure 3). This involved rotating each traction force map by the positive angle separating the velocity vector and the negative x-axis, which we chose as the positive direction of travel for any arbitrary growth cone. After rotation, traction force maps were cropped and resampled appropriately to preserve the original scale and resolution of the pre-rotated image. After rotation, the resulting traction force maps for each dataset were added together to create an average picture of the spatial distribution of tractions forces generated in the inertial reference frame of the growth cone over the timespan of the dataset (Figure 4).

The nature and morphology of the growth cones were often highly dynamic. Specifically, the position and direction of movement as obtained from a function of the spatial distribution of traction forces (described above) could vary greatly on small timescales compared to the the directional variation of the axon trajectory characterized over larger time frames. Thus, on short timescales, instantaneous displacement produced large variations relative to the long-term behavior of the growth cone, so we imposed a discrete moving-window smoothing function on *R* for each dataset. The size of the window was determined as a function of Δ*t* to account for the variation in observation time resolutions across the datasets considered for this analysis. A further measure was taken to account for periods where the growth cone “stalled” or ceased forward movement. If the dot product of the velocity vector and its successor was less than zero, those time points were removed from consideration due to the fact that our analyses focused only on the general forward movement of the axon and growth cone. Explicitly, if for any consecutive time points *n*, *n*+1,

(3) $${\vec{v}}_{n}\cdot {\vec{v}}_{n+1}<0,$$

the traction force maps associated with those time points were not included in our analysis for that particular dataset.

Stress peak tracking was done using Imaris (Bitplane, Zurich) particle tracking software. Stress peaks were initially detected and tracked automatically using a region-growing detection algorithm, where a peak A in one frame was associated with a peak B in the next frame if both the distance between A and B was small enough and if B was the closest to A of all peaks detected in the second frame. Tracks were then manually hand checked and edited for each dataset to account for errors due to algorithmic limitations (Supplementary Video 1).

We calculated the autocorrelation,

$$R(\tau )=\frac{<[(F(t)-\mu )(F(t+\tau )-\mu )]>}{\sigma^{2}},$$

for the net force time series for each high-time resolution dataset, where τ was the time separation, σ is the standard deviation of the time series, μ is the average of the time series, and each sum is over all time *t*. We considered lag times, or time separation, τ up to 480 s, and normalized the result for each dataset by its mean (**Figure 7**).

We also calculated the mean squared displacement of the net force,

$$MSD(\tau )=<{\left[F(t+\tau )-F(t)\right]}^{2}>,$$

for each high-time resolution dataset, where τ was the time separation, and each sum is over all time *t*. We considered values of τ to 900 (s), half the duration of the capture lengths of the datasets, and normalized the result for each dataset by its mean (**Figure 8**).

The traction force calculation and analysis code used in the methods described above may be found at https://github.com/rjpolackwich/gctf.

## 4. Results

### 4.1. Determination of average growth cone stress field

We have previously reported that growth cone traction stresses for developing rat DRG neurites display complex spatiotemporal patterns, with maximum stresses typically appearing in the peripheral region between the central zone close to the axon neck and the filopdia at the distal end of the axon (Koch et al., 2012). At any particular instant, a snapshot of that stress field appears quite disordered, although the peak stresses are usually directed toward the central zone, and the integrated net force was found to be parallel with the axon, as required by force balance.

In order to determine the average behavior produced by the fluctuating stress field, we developed an algorithm for determining growth cone positions directly from the stress field. We observed that, since the traction stresses are consistently distributed around the DRG growth cone, they provide a “footprint” that reliably encompasses the center of the growth cone. As described in Section 3 and shown in Figure 1, we used the center of the convex hull of this footprint to algorithmically extract growth cone positions from each stress map. Comparisons with cell outlines from images taken concurrently with traction force microscopy confirmed that the position determined by this method provided a reasonable estimate for the middle of the growth cone (Supplementary Video 2).

Using the resulting series of growth cone positions (Figure 2), we transformed each stress field to a coordinate system fixed on the advancing growth cone (Figure 3), as described in Section 3. Averaging the stress fields from the entire time series for the advancing growth cone produced a remarkably clear and consistent symmetric pattern, one example of which is shown in Figure 4. The average stress field has strong maxima on the sides of the growth cone, directed inward toward the growth cone neck. This pattern is reminiscent of the “force dipoles” observed in isolated cells (Mandal et al., 2014; Tanimoto and Sano, 2014), but with a residual unbalanced net force, indicative of the axonal tension. We performed this analysis on 10 datasets of DRG trajectories across a range of relatively soft (150–400 Pa) polyacrylamide substrates, captured at time intervals ranging between 120 and 180 s. We found that the average traction force pictures were similar across all substrate stiffness studied (Supplementary Figure 1).

### 4.2. Localized stress peak lifetimes

The traction stresses arise from dynamic stress-bearing adhesions that form and dissipate on timescales too short to be measured with the low temporal resolution of the data described above and reported in Koch et al. (2012). Therefore, we performed a series of traction force measurements with much higher time resolution, with an image every 2 s. This procedure required subjecting the cells to nearly continuous illumination, and many neurites retracted in response, but we were able to record 5 data sets from steadily advancing growth cones. For these data sets, the rise and fall of localized traction stress maxima could be clearly resolved (See Section 3 and Supplementary Video 1).

**Figure 5:**
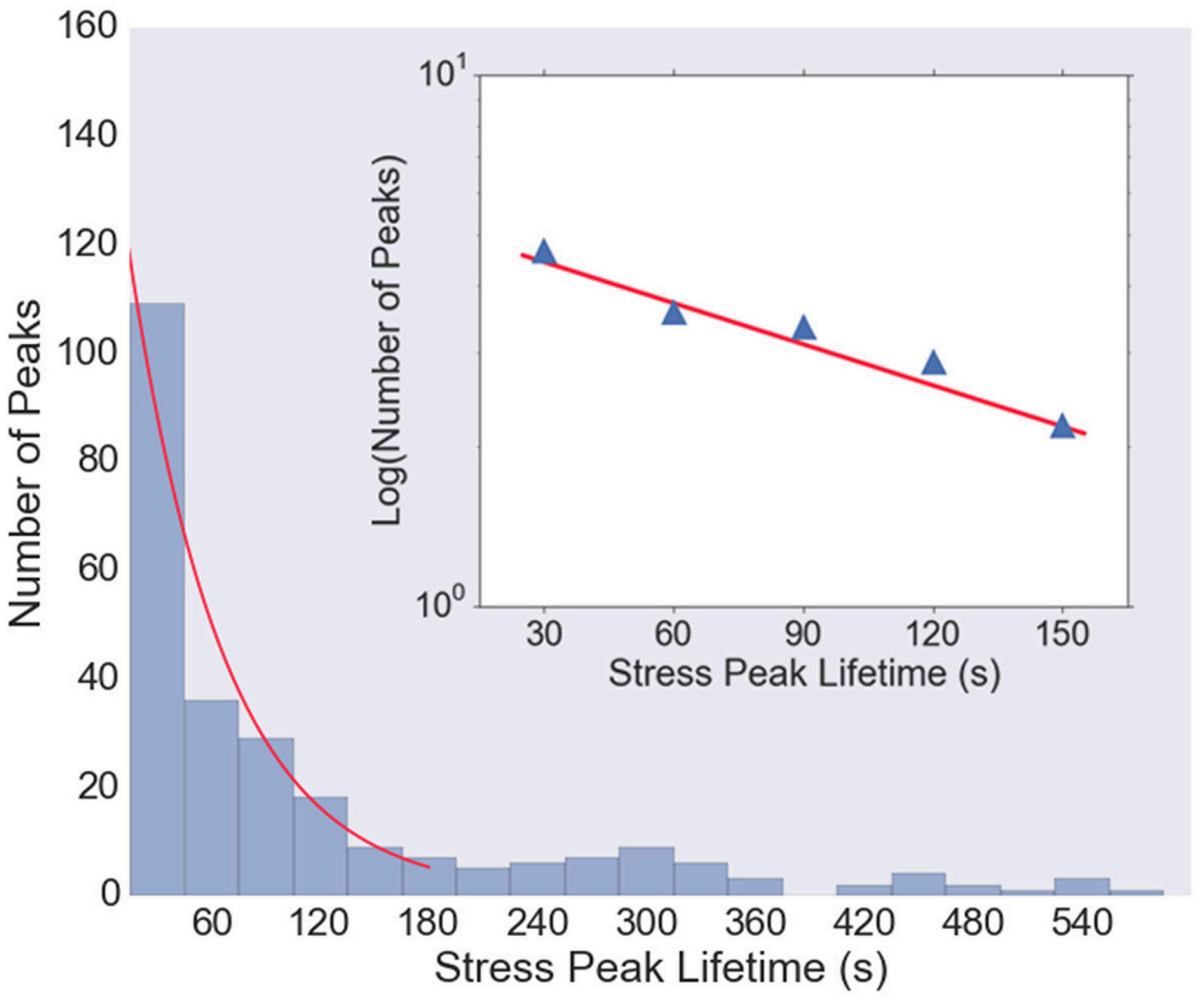
**Histogram of lifetimes of stress peaks**. Most stress peaks are of short duration, with some longer, and peak lifetimes of about 30–150 s are well-fit with an exponential distribution (red curve). Very short (< 15 s) and very long (>600 s) lived stress peaks are not shown in the histogram. Inset: Log-linear plot of histogram values from *t* = 30 to *t* = 150 s, with a least-squares fit (red line) with slope 1/38 s, indicating that the majority of the stress peak lifetimes follow a Poisson distribution with a decay time of about 38 s.

Figure 5 shows the histogram of stress peak lifetimes extracted from all five data sets. There are a large number of very short-lived peaks, followed by an intermediate regime that is well-described by a Poisson distribution (an exponential decay) characterized by a decay time of 38 s (Figure 5, inset), followed by a long tail of very long-lived peaks. The length of the trajectories (1800 s) is sufficiently long that the statistics in the exponentially decaying region are be significantly affected by the finite observation time.

### 4.3. Neurite tension fluctuations

**Figure 6:**
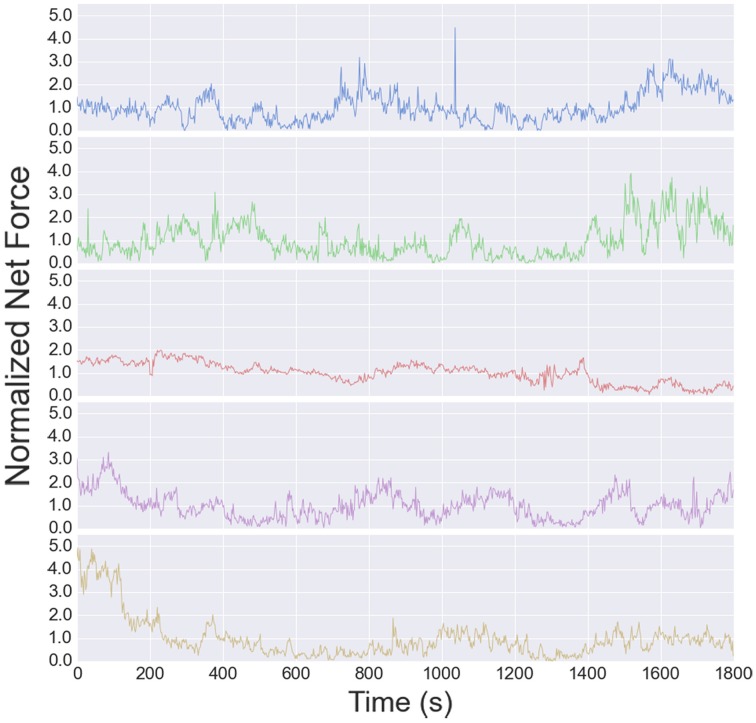
**Net tension vs. time for each growth cone used for the fluctuation analysis**. The fluctuation size is larger than the mean, resulting in a negative autocorrelation at long times (Figure 7).

The traction stress maps provide a direct, dynamic readout of neurite tension. As reported in Koch et al. (2012), the tension in DRG neurites varies over a modest range, and is independent of substrate stiffness over the accessible range (see Supplementary Figure S6B in Koch et al., 2012). The high time resolution of the data analyzed here provides a much more detailed picture of the tension dynamics. The time series for all 5 cells are shown in Figure 6. The tension appears to vary randomly over short time scales, although the change from one image to the next is usually small, indicated that measurement noise does not contribute significantly to the fluctuations (each stress map is computed independently).

**Figure 7:**
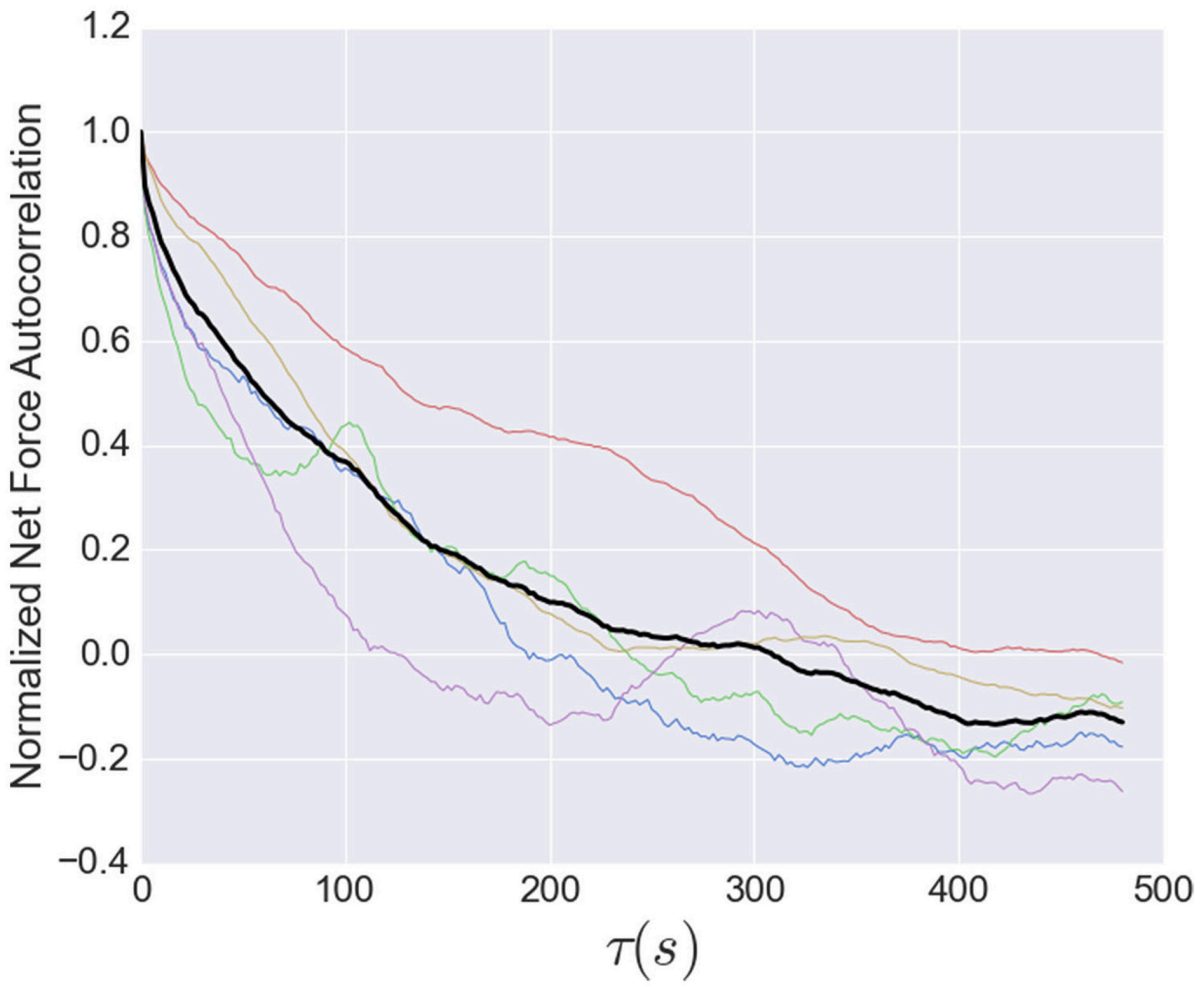
**Normalized net force autocorrelation**. The fluctuations in the tension (net force) are shown for each dataset (colored lines) up to lag times of 500 s. The black line represents the ensemble average. Fluctuations from one image to the next are small, however, the correlations decay relatively quickly, with an initial rapid drop followed by a roughly exponential decay from 10 to 80 s with a decay time of 95 s, indicating that tension fluctuations at larger times are mostly uncorrelated. This decay time is a little more than twice that of decay time for the exponential region of the stress peak lifetime distribution (Figure 5).

The timescale for the tension fluctuations can be quantitatively determined through the calculation of the autocorrelation function, shown in Figure 7. This standard analysis for stochastic signals shows the extent to which the variations in the tension at one time are correlated with those at a later time. The correlation at very short times is near unity, indicating the fluctuations from one image to the next are small, as stated above. The correlations decay relatively quickly, however, with an initial rapid drop followed by a region from 10 to 80 s that is well-described by an exponential decay with a decay time of 95 s, indicating that tension fluctuations over times much larger than this are mostly uncorrelated. This decay time is a little more than twice that of decay time for the exponential region of the stress peak lifetime distribution (Figure 5).

**Figure 8:**
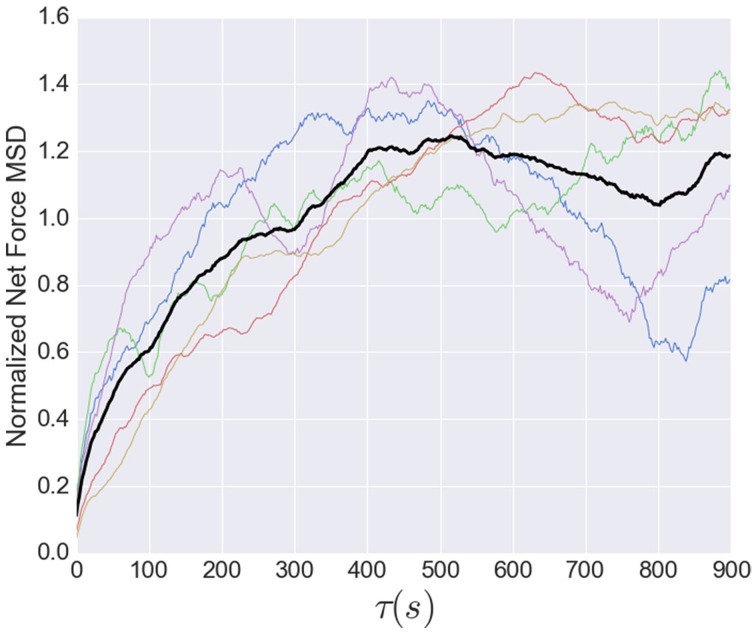
**Normalized mean squared displacement of net force fluctuations**. The colored lines represent the individual MSD's, while the black line shows the average of all five. Each dataset was normalized by its average. The MSD is bounded (as expected because the growth cone's net force is limited) and grows sub-diffusively, with an apparent exponent of approximately 3/7.

While the short time dynamics of the tension fluctuations appears stochastic, the tension cannot grow without bound. The total traction stress generated across the growth cone is nearly constant (Koch et al., 2012), which may be related to the relatively constant overall growth cone size. The effect of this constraint on the tension fluctuations can be seen in the growth of the mean squared displacement (MSD) over time, show in Figure 8. This analysis, often used for trajectories of diffusing particles, describes the average difference between tension values separated by a time τ. The MSD grows rapidly at short times, as expected by the stochastic fluctuations evidenced in the autocorrelation function. However, the MSD saturates after ~400 s, indicating that the range of fluctuations is constrained. The MSD curves in Figure 8 are normalized to the average tension, and the fact that the saturation occurs at a value greater than unity is a consequence of the large fluctuations in tension relative to the average (Figure 6). The overall shape of the MSD curve is thus consistent with diffusive motion within a bounded region, but the curvature is much stronger than for normal diffusion. In fact, from 2 to 200 s the MSD grows roughly as τ^3/7^, compared to an exponent of one for normal diffusion. However, given the substantial cell-to-cell variability observed, significantly more data will be required before strong quantitative conclusions can be drawn about the average population statistics.

## 5. Discussion

We have shown that there is a clear, robust pattern underlying the complex spatio-temporal dynamics of traction stresses generated by the growth cones of advancing rat DRG axons. The pattern is reminiscent of the force dipoles observed in isolated motile cells, which typically arise from the effects of myosin motors generating tension in actin stress fibers anchored at adhesion sites at opposite sides of a cell (Mandal et al., 2014; Tanimoto and Sano, 2014). Unlike isolated cells, however, traction stresses within the growth cone are not balanced, and instead have a net force that is equal to the tension in the axon. A similar situation is observed in the much larger growth cones of *aplysia* bag cell neurons, but with significantly reduced temporal variation (Hyland et al., 2014). On the other hand, the much smaller growth cones of rat hippocampal axons produce intermittent, localized traction stresses that are always parallel with the axon. One straightforward explanation for this range of behaviors is that the size of the growth cone determines the number of independently fluctuating adhesion sites that are present in the growth cone at any instant. If there is only one, tension can only be generated between that site and the axon. When there are multiple sites, tension can be generated both between adhesion sites and between the adhesions and the axon. For large growth cones, the number of adhesion sites may be large enough that the fractional fluctuations are relatively small, and the stress pattern is directly related to the relatively constant distribution of adhesion sites.

Using traction stress measurements with very high time resolution, we were able to characterize the lifetimes of individual sites of traction stress. We find that there are a large number of very short-lived sites, and a roughly equal number that are distributed in a Poisson distribution, with a characteristic decay time of 38 s. This is roughly consistent with the timescale reported for traction stress peaks in *aplysia* growth cones (Hyland et al., 2014) and for growth cone adhesion sites (Woo and Gomez, 2006), although significantly longer than the timescale of ~10 s reported for fluctuations in protrusive activity at the leading edge of the growth cone (Betz et al., 2007). In addition, some growth cones also display very long-lived adhesion sites that translate as they advance, producing large axon tension.

The high time-resolution measurements show remarkable fluctuations in the net force, representing the axon tension. To our knowledge, this is the first report of this phenomenon. The autocorrelation of the fluctuations suggests an underlying stochastic component that varies on a timescale of tens of seconds, but that the tension does not fluctuate too far from its long-time average. The most likely source of the tension fluctuations is the formation and disassembly of adhesions, as discussed above. The similarity in timescales between local stress maxima lifetimes and overall tension fluctuations supports this simple picture. However, fluctuations in active tension generation in the axon may play a role as well.

Mature axons, where the growth cone is no longer present, exhibit a resting tension, and their response to perturbations can be modeled by active contraction and viscoelastic relaxation (Bernal et al., 2010). Recent results from force measurements on towed axons are consistent with contractile forces originating both from the rear of the growth cone and the axon itself, as well as viscous dissipation from the flow of cytoskeletal components (O'Toole et al., 2015). Thus, a reasonable minimal model combining these results would include active force generation in the axon and the base of the growth cone, as well as in the growth cone itself (to generate the force-dipole component, as well as a component parallel to the axon), with fluctuations arising solely from the adhesion dynamics. In this picture, the myosin-dependent contractility that is necessary for traction stress generation (Koch et al., 2012) produces an essentially constant contractile stress (and a roughly constant rate of actin retrograde flow in the growth cone), and the crucial dynamics are those of the adhesions and their connection to the growth cone cytoskelton. The stochastic adhesion dynamics may provide a mechanism for probing substrate stiffness and thus enable mechanical guidance (durotaxis), and can be sensitively modulated during chemical guidance (chemotaxis).

The work presented here is limited to a small number of growth cones, and restricted to those that are steadily advancing. Future studies should include a wider range of behaviors, in particular pausing, turning, and branching, as these are essential for understanding axon guidance and neuronal maturation. In addition, high time-resolution traction force measurements on other neuronal cell types are necessary to determine if the picture presented can be applied to neurons with small growth cones showing highly intermittent tension, as seen in rat hippocampal neurons (Koch et al., 2012), and those with large growth cones showing nearly constant tension, as observed in *aplysia* growth cones (Hyland et al., 2014). Finally, experimental manipulations of cytoskeletal components are required to illuminate the underlying mechanisms of force generation within the growth cone and its role in axon growth and guidance.

## Author contributions

JP performed the data analysis presented in the manuscript, DK performed all experiments and initial analyses, RM assisted with all phases of the project, and HG and JU supervised the work. JP, RM, and JU generated the manuscript, with input from DK and HG.

### Conflict of interest statement

The authors declare that the research was conducted in the absence of any commercial or financial relationships that could be construed as a potential conflict of interest.

## Supplementary material

Supplementary Figure 1 **Average spatial traction force distributions exerted by 9 different growth-cones, as described by Figure 4**. We observed a consistent “force dipole” pattern across a range of substrate stiffnesses. Each label refers to the value of the Young's modulus of the substrate (in Pascal). Scale bars represent 5 microns.

Supplementary Video 1 **Example automatic peak tracking results**. The location of identified stress peaks are marked with red spheres. Obvious gaps in stress peak trajectories were manually corrected after automatic tracking to account for algorithmic errors.

Supplementary Video 2 **Tracking of a growth cone over time**. A growth cone is tracked using the traction stress convex hill centroid method as described in Methods for a fluorescently labeled axon (actin-GFP transfection as described in Koch et al., 2012).
