# Supplementary figures and images for: Traction force and tension fluctuations in growing axons
Source: Front Cell Neurosci. 2015 Oct 29;9:417. doi: 10.3389/fncel.2015.00417 (PMC4624864; doi:10.3389/fncel.2015.00417)

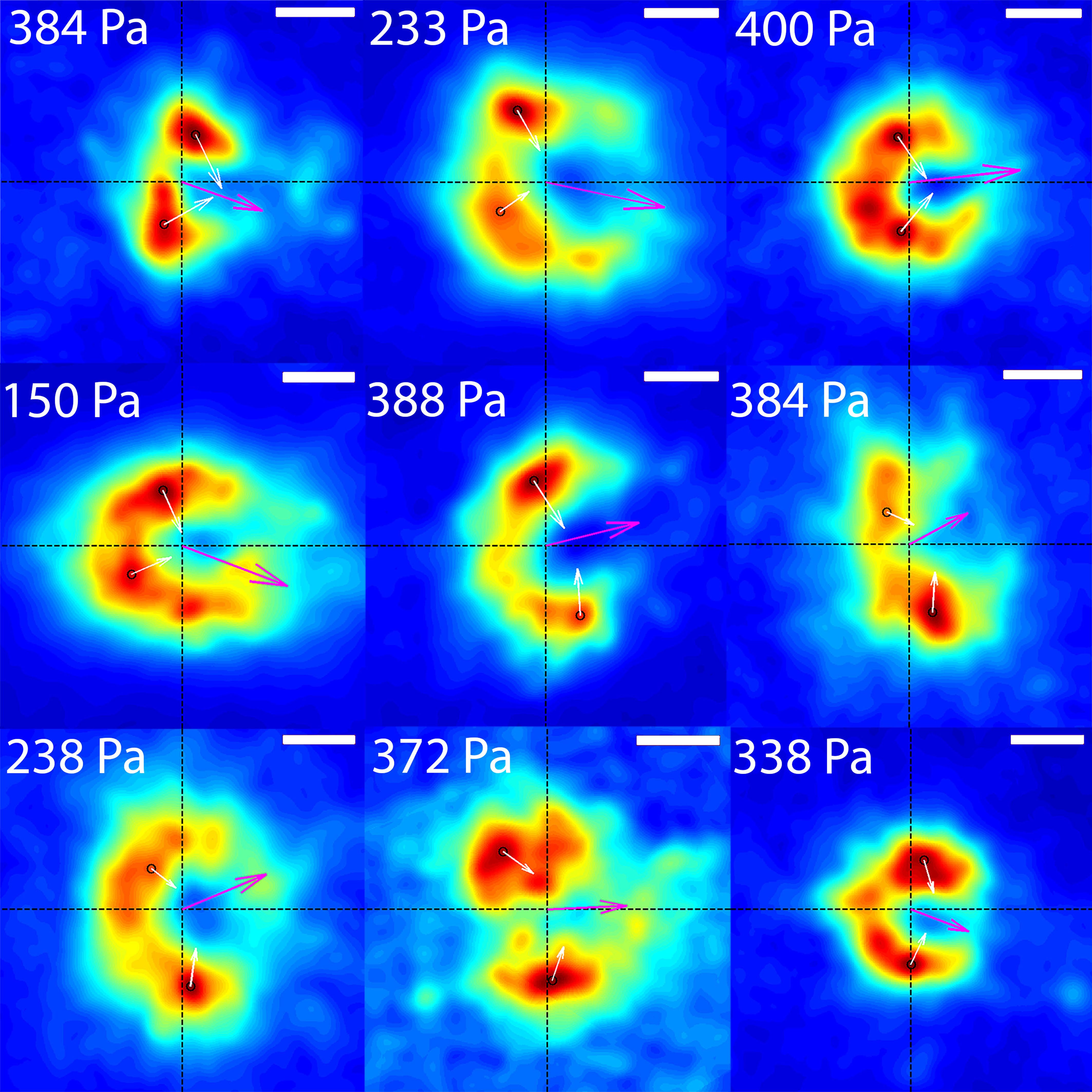

Supplement: Supplementary Figure 1 — Average spatial traction force distributions exerted by 9 different growth-cones, as described by Figure 4. We observed a consistent “force dipole” pattern across a range of substrate stiffnesses. Each label refers to the value of the Young's modulus of the substrate (in Pascal). Scale bars represent 5 microns. [file Image1.JPEG]
